# Cold Atmospheric Plasma Modification of Amyloid β

**DOI:** 10.3390/ijms22063116

**Published:** 2021-03-18

**Authors:** Maho Yagi-Utsumi, Tomohiro Tanaka, Yoko Otsubo, Akira Yamashita, Shinji Yoshimura, Motohiro Nishida, Koichi Kato

**Affiliations:** 1 Exploratory Research Center on Life and Living Systems (ExCELLS), National Institutes of Natural Sciences, Okazaki, Aichi 444-8787, Japan; mahoyagi@ims.ac.jp (M.Y.-U.); ttanaka@nips.ac.jp (T.T.); nishida@nips.ac.jp (M.N.); 2 Institute for Molecular Science (IMS), National Institutes of Natural Sciences, Okazaki, Aichi 444-8787, Japan; 3 National Institute for Physiological Sciences, National Institutes of Natural Sciences, Okazaki, Aichi 444-8787, Japan; 4 Department of Plasmabio Science, Center for Novel Science Initiatives, National Institutes of Natural Sciences, Minato-ku, Tokyo 105-0001, Japan; otsubo@nibb.ac.jp (Y.O.); ymst@nibb.ac.jp (A.Y.); yoshimura.shinji@nifs.ac.jp (S.Y.); 5 National Institute for Basic Biology, National Institutes of Natural Sciences, Okazaki, Aichi 444-8585, Japan; 6 National Institute for Fusion Science, National Institutes of Natural Sciences, Toki, Gifu 509-5292, Japan; 7 Center for Low-temperature Plasma Sciences, Nagoya University, Nagoya 464-8601, Japan

**Keywords:** amyloid β, cold atmospheric plasma, hydrogen peroxide, NMR

## Abstract

Cold atmospheric plasma (CAP) has attracted much attention in the fields of biotechnology and medicine owing to its potential utility in clinical applications. Recently accumulating evidence has demonstrated that CAP influences protein structures. However, there remain open questions regarding the molecular mechanisms behind the CAP-induced structural perturbations of biomacromolecules. Here, we investigated the potential effects of CAP irradiation of amyloid β (Aβ), an amyloidogenic protein associated with Alzheimer’s disease. Using nuclear magnetic resonance spectroscopy, we observed gradual spectral changes in Aβ after a 10 s CAP pretreatment, which also suppressed its fibril formation, as revealed by thioflavin T assay. As per mass spectrometric analyses, these effects were attributed to selective oxidation of the methionine residue (Met) at position 35. Interestingly, this modification occurred when Aβ was dissolved into a pre-irradiated buffer, indicating that some reactive species oxidize the Met residue. Our results strongly suggest that the H_2_O_2_ generated in the solution by CAP irradiation is responsible for Met oxidation, which inhibits Aβ amyloid formation. The findings of the present study provide fundamental insights into plasma biology, giving clues for developing novel applications of CAP.

## 1. Introduction

Cold atmospheric plasma (CAP) has been widely used across the industrial fields as exemplified by sterilization and surface fabrication. Recently, CAP has attracted much attention in the biotechnology and medical fields [1,2]. Various studies have been conducted on the effect of non-thermal plasmas for wound healing, blood coagulation, skin regeneration, and action against cancer cells [3]. Therefore, it is of great interest and importance to elucidate how the structures, motions, and associations of biomacromolecules are affected by plasma irradiation from the perspective of plasma biology and biomolecular science. In a recent series of pioneering studies, Lee and coworkers investigated the effects of CAP on the biomolecular structures using various globular proteins, including myoglobin, hemoglobin, and lysozyme, as well as amino acids, as model molecules [4,5,6,7]. These data demonstrated that carbonylation occurs in protein due to reactive oxygen and nitrogen species generated by CAP, which induces changes in the secondary and/or tertiary structures of proteins. This is exemplified by the structural perturbation of lysozyme coupled with a modification of the tryptophane group in its substrate binding site [5]. In addition, plasma-induced chemical modifications of amino acids have been reported, including hydroxylation and nitration of aromatic rings in tyrosine, phenylalanine, and tryptophan, sulfonation and disulfide linkage formation of thiol groups in cysteine, sulfoxidation of methionine, and amidation and ring-opening of five-membered rings in histidine and proline [4,8]. In addition, Bayliss et al. reported that amyloid fibrils can be degraded by CAP treatment [9]. Although these CAP effects have been ascribed to some chemical modifications, there remain open questions regarding the molecular mechanisms behind the observed phenomena.

Considering its clinical importance, the molecular mechanisms underlying the assembly of amyloid-β (Aβ), which is associated with Alzheimer’s disease, have been extensively investigated through NMR spectroscopy, electron microscopy, computational simulation, and other biophysical techniques [10,11,12,13,14,15,16,17,18,19,20]. These studies indicated that the Aβ amyloid formation processes and the consequent fibril morphology are strongly influenced by amino acid sequence modifications, such as familial mutations, as well as by solution environments, which includes ionic strength, pH, temperature, pressure, and gravity as well as membrane environments. It is therefore intriguing to address the mechanisms of the effects of CAP on the Aβ assembly.

In this study, we performed structural and kinetic analyses of amyloid fibril formation of the CAP-treated Aβ protein. We attempted to identify Aβ structural modifications caused by CAP irradiation and have discussed the possible mechanisms of the effects of CAP on amyloid formation.

## 2. Results and Discussion

### 2.1. NMR Characterization of CAP-Treated Aβ

**Figure 1:**
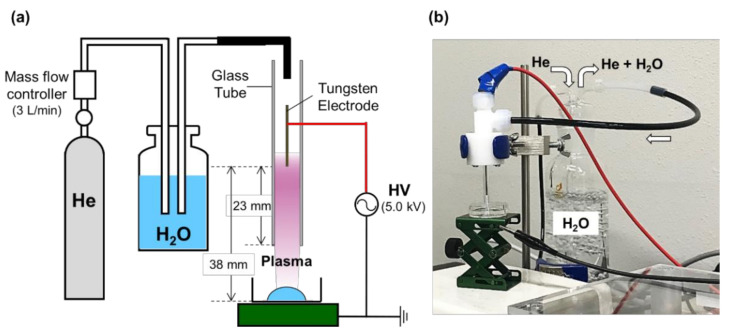
(**a**) Schematic illustration and (**b**) picture of the experimental setup for the helium atmospheric pressure plasma irradiation system primarily used in this study. In this 8.0 W plasma jet system, He gas passed through pure water and moist He with 100% relative humidity served as the source gas. The applied peak-to-peak voltage (5.0 kV), the distance between the electrodes (38 mm), and the distance between the powered electrode and the edge of the glass tube (23 mm) were represented.

**Figure 2:**
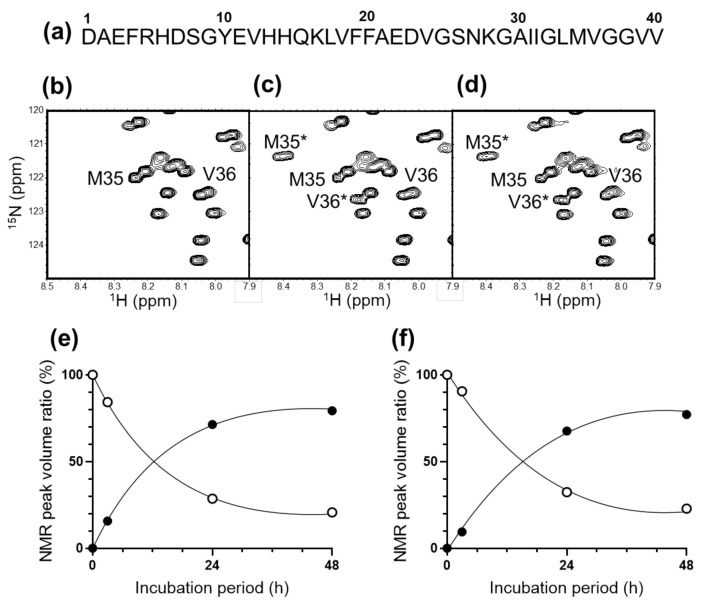
NMR spectral change of cold atmospheric plasma (CAP)-treated Aβ (1–40). (**a**) The primary structure of Aβ (1–40) peptide. (**b**) A portion of ^1^H–^15^N heteronuclear single-quantum correlation (HSQC) spectra of Aβ (1–40) without irradiation. Portions of ^1^H–^15^N HSQC spectra of Aβ (1–40) measured at 48 h (**c**) after a 10 s irradiation of CAP or (**d**) after dissolving into the 10 s pre-irradiated buffer solution. The time course of NMR peak volume ratio of Met35 (^1^H 8.2 ppm, ^15^N 122.0 ppm, open circle) and Met35* (^1^H 8.4 ppm, ^15^N 121.4 ppm, closed circle) of Aβ (1–40) (**e**) after the 10 s irradiation of CAP or (**f**) after dissolving into the pre-irradiated buffer solution.

We first examined the possible effects of CAP irradiation on Aβ (1–40) structure. The Aβ (1–40) solution was irradiated with moistened helium gas for 10 s using a plasma source (Figure 1). The structural changes before and after plasma irradiation were tracked using ^1^H–^15^N heteronuclear single-quantum correlation (HSQC) peaks as spectroscopic probes (Figure 2 and Supplementary Materials Figure S1). Although no significant spectral change was observed immediately after CAP irradiation, the peaks originating from the segment from Ile32 to Val39, especially those from Met35 and Val36, gradually exhibited significant chemical shift changes after incubation at 37 °C (i.e., from Met35 to Met35* and from Val36 to Val36*) (Figure 2c). The magnitudes in the spectral changes gradually reached a saturation point after the CAP irradiation and did not return to the original peak position (Figure 2c,e).

Similar spectral changes were also observed when Aβ (1–40) was dissolved into the buffer solution immediately after buffer pretreatment with CAP (Figure 2d,f). These observations suggest that the gradual structural modification of Aβ (1–40) was not a consequence of direct plasma–protein interaction; rather, it was caused by some reactive species generated in the solution upon CAP irradiation.

### 2.2. CAP Effects on Aβ Fibril Formation

**Figure 3:**
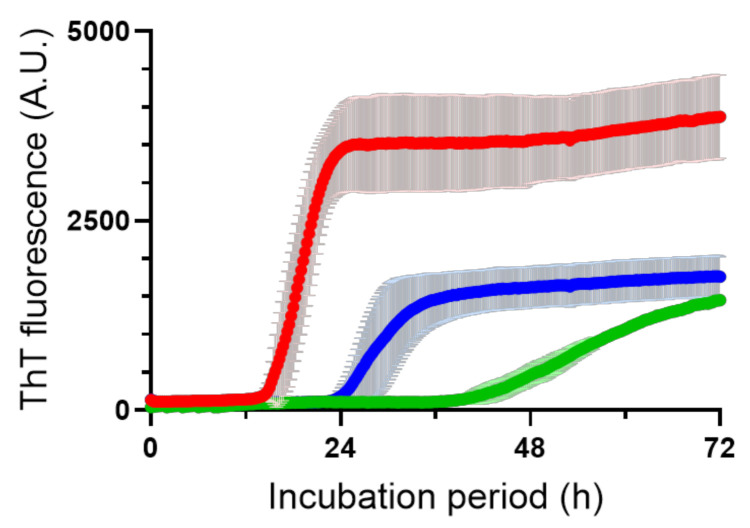
ThT fluorescence intensity profiles of the aggregation of Aβ (1–40) without (red) and with CAP pretreatment for 10 s (blue) or 20 s (green). Each intensity value is the mean ± SD of three values.

We next examined whether plasma irradiation has any effects on Aβ (1–40) fibril formation by investigating the time course of fibrilization with or without pretreatment of Aβ (1–40) solution with CAP for 10 s. The fibril growth was probed with the thioflavin T (ThT) dye, which specifically binds and colors amyloid fibrils. We noted that the plasma irradiation resulted in considerably slower progress in Aβ (1–40) fibrilization (Figure 3). Generally, the amyloid fibrilization process consists of the nucleation and elongation phases. We noted that nucleation was delayed and that the fibril elongation rate also slowed down with increasing CAP irradiation time, which resulted in lower final fibril yield. These kinetic data suggest that the pre-irradiated Aβ (1–40) formed significant amounts of non-fibril aggregates compared with the non-irradiated ones. We confirmed that the CAP irradiation suppresses the fibrilization of Aβ (1–42) as well (Figure S2).

### 2.3. Characterization of Chemical Modification of Aβ Caused by CAP Treatment

**Figure 4:**
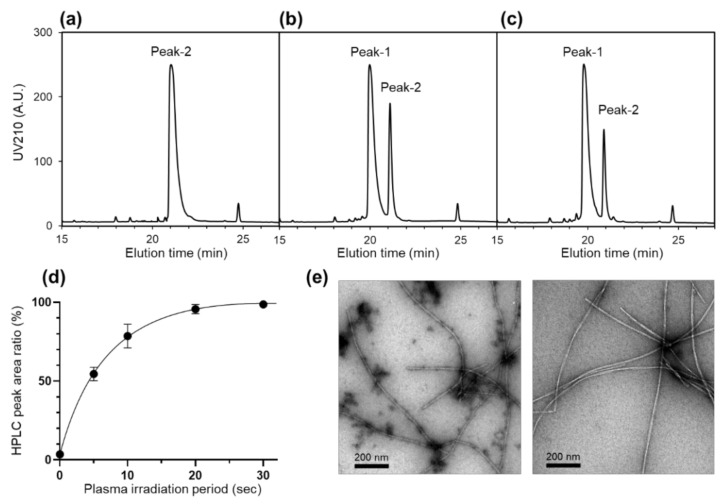
HPLC profiles of (**a**) Aβ (1–40) without CAP treatment, (**b**) Aβ (1–40) with 10 s CAP treatment, and (**c**) Aβ (1–40) dissolved into the buffer solution pretreated with CAP. (**d**) Changes in the HPLC peak area ratio of peak-1 to peak-2 depending on the period of CAP irradiation. Each ratio value is the mean ± SD of three values. (**e**) TEM images of amyloid fibrils prepared by Aβ (1–40) in peak-1 (left panel) and in peak-2 (right panel) fractions.

**Figure 5:**
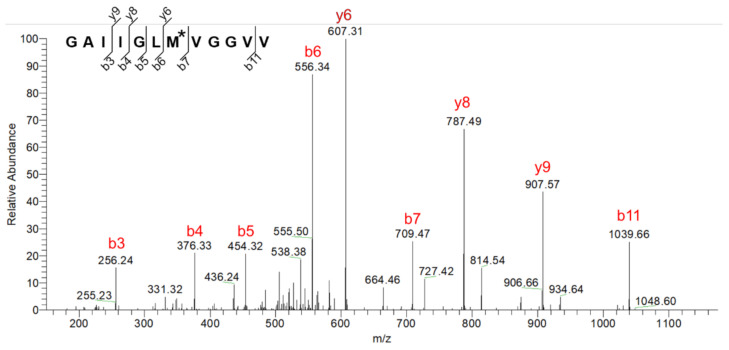
LC-MS/MS fragmentation profile of tryptic peptide of Aβ (1–40) fraction corresponding to peak-1. M* denotes oxidized Met.

To identify the chemical modifications associated with the observed perturbation on Aβ structure and assembly, the CAP-treated Aβ (1–40) solutions were subsequently applied to reverse-phase HPLC and then analyzed by MALDI-TOF-MS and LC-MS/MS. In the HPLC profiles, the 10 s plasma treatment gave rise to a new peak (peak-1) that eluted at 20.0 min with a concomitant reduction in the original peak (peak-2) at 21.1 min (Figure 4a–c). Aβ (1–40) dissolved into the pre-irradiated buffer solution also exhibited peak-1 and peak-2 with a ratio of 8:2. Longer CAP irradiation resulted in an increase in the peak-1 intensity with a concomitant decrease in the peak-2 intensity (Figure 4d). MALDI-TOF-MS data showed that the molecular weights of Aβ (1–40) proteins corresponding to peak-1 and peak-2 were 4592.6 and 4576.6, respectively. Furthermore, the LC-MS/MS analysis revealed that the mass of Met35 increased by +16.0 upon CAP irradiation (Figure 5 and Supplementary Materials Table S1). These results indicated that the plasma irradiation resulted in selective oxidization of this methionine residue into methionine sulfoxide.

Based on the ^1^H–^15^N HSQC spectral data of the Aβ (1–40) species corresponding to peak-1 and peak-2 (Figure S3), we confirmed that the aforementioned spectral changes, along with the changes in the fibril formation kinetics, caused by CAP treatment were ascribed exclusively to the formation of sulfoxide of Met35. Although transmission electron microscope (TEM) observation revealed no significant morphological difference in the mature fibrils formed from the Aβ(1–40) species corresponding to the peak-1 and peak-2 fractions, significant amounts of non-fibril aggregates were observed only in the specimens from the peak-1 fraction (Figure 4e). The accumulation of non-fibril aggregates is consistent with the lower level of final fibril yield, as probed with ThT (Figure 3). These data indicate that the Met35 oxidation prevented fibril formation.

### 2.4. Reactive Species Responsible for Methionine Oxidation of Aβ

**Figure 6:**
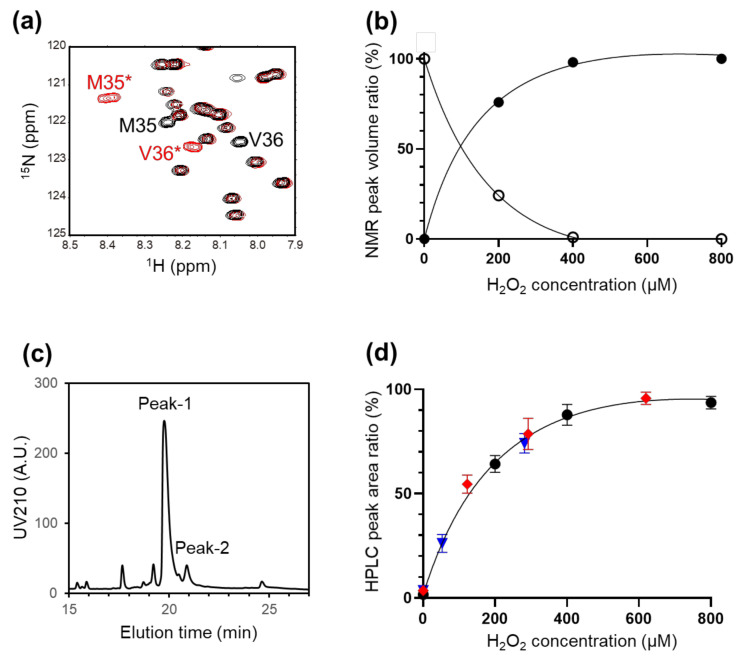
(**a**) The portions of ^1^H–^15^N HSQC spectra of Aβ (1–40) in the absence (black) and presence (red) of 800 μM H_2_O_2_. The spectrum of Aβ (1–40) was measured at 24 h after H_2_O_2_ treatment. (**b**) Changes in the NMR peak volume ratio of Met35 (^1^H 8.2 ppm, ^15^N 122.0 ppm, open circle) and Met35* (^1^H 8.4 ppm, ^15^N 121.4 ppm, closed circle) of Aβ (1–40) depending on the H_2_O_2_ concentration. (**c**) HPLC profiles of Aβ (1–40) treated with 800 μM H_2_O_2_. (**d**) Changes in the HPLC peak area ratio of peak-1 to peak-2 depending on the H_2_O_2_ concentration. HPLC peak area ratios were plotted as a function of the H_2_O_2_ concentration used for the Aβ (1–40) treatment (black circle) along with those from the CAP irradiation experiments. For plotting, the CAP irradiation period was converted into the concentration of H_2_O_2_ generated in the buffer solution pretreated with CAP using 8.0 W and 1.0 W plasma jet apparatuses (according to the results shown in Figure S4 (red rhombus) and Figure S5 (blue triangle), respectively). Each ratio value is the mean ± SD of three values.

We attempted to identify the putative reactive species causing the methionine oxidation of Aβ (1–40). In fact, in the presence of 1 mM ascorbic acid as a reducing reagent, virtually no HSQC spectral changes were observed for a CAP-treated Aβ (1–40) solution (Figure S1d). Various reactive oxygen species, including H_2_O_2_, are supposed to be produced upon water irradiation with CAP [21,22]. In the present study, we confirmed that H_2_O_2_ is generated by CAP irradiation in the buffer solution (Figures S4 and S5). We thus measured the ^1^H–^15^N HSQC spectra of Aβ (1–40) in the presence of varying concentrations of H_2_O_2_, thereby demonstrating similar spectral changes to those observed in the Aβ (1–40) solution pre-irradiated with CAP (Figure 6a,b). Based on the HPLC profiles, the levels of Met35 oxidation were quantified as a function of H_2_O_2_ concentration (Figure 6c,d). We also estimated the relationship between the Met35 oxidation level and the concentration of H_2_O_2_ generated by CAP irradiation using the two different apparatuses (Figure 6d). These data are quantitatively in good agreement. In addition, the NMR, TEM, and kinetics outcomes are largely consistent with the previously reported observations for H_2_O_2_-treated Aβ proteins [23,24]. All these data cumulatively suggested that CAP-generated H_2_O_2_ induces methionine oxidation of Aβ (1–40). As exemplified by its shorter retention time, Aβ (1–40) with oxidized Met35 has less hydrophobic nature, which may hamper its fibrilization (Figure 4b,c). Previously reported molecular dynamics simulation demonstrated that an Aβ pentamer is destabilized upon oxidation of Met35 due to lower inter-peptide binding free energy, resulting in a higher conformational flexibility with increasing solvent accessibility [25]. Indeed, we confirmed that the Aβ (1–40) fibrils were degraded by incubation in the CAP-pretreated buffer as in the case of H_2_O_2_ treatment (Figure S6). Considering the findings obtained in this study, CAP-generated H_2_O_2_-induced degradation of Aβ aggregates can also be ascribed to the Met 35 oxidation.

## 3. Materials and Methods

### 3.1. Aβ Sample Preparation

Bacterial expression and purification of isotopically labeled Aβ (1–40) protein was performed as described previously [11]. Synthetic Aβ (1–42) peptide was purchased from Toray Research Center, Inc. (Tokyo, Japan). Aβ (1–40) was dissolved at an approximate concentration of 2 mM in 0.1% (*v*/*v*) ammonia solution, followed by their collection and storage in aliquots at −80 °C until further use. Amyloid fibrils were prepared by incubation of 0.1 mM [U-^15^N] Aβ (1–40) at 37 °C for 3 days in 10 mM potassium phosphate buffer (pH 7.4).

### 3.2. CAP Irradiation

We performed irradiation of He atmospheric cold plasma using two different laboratory-built apparatuses [26,27,28]. [U-^15^N] Aβ (1–40) was dissolved at a concentration of 0.1 mM in 10 mM potassium phosphate buffer (pH 7.4). In the apparatus primarily used in this study, the calculated mean input power was 8.0 W and moist He with 100% relative humidity served as the source gas, with a flow rate of 3 L/min through the dielectric tube regulated by a mass flow controller [26,27]. When the timer-controlled high-voltage power supply (with a frequency of 10 kHz) of this system was turned on, He atmospheric cold plasma was generated and it flowed out from the nozzle of the glass tube of 2 mm diameter (Figure 1). In the other apparatus, the calculated mean input power was 1.0 W, and He gas was supplied with a flow rate of 3 L/min through a copper pipe attached to the Peltier device (LVPU-30; VICS, Tokyo, Japan) [28]. The generated plasma was applied for irradiation to Aβ (1–40) or buffer solution in a glass-base dish for 10–30 s. A 250 μL aliquot in a φ12-mm dish (IWAKI, Fukushima, Japan) was subjected to the 8.0 W plasma jet at room temperature, whereas a 200 μL aliquat in a φ10 mm dish (MatTek, Ashland, MA, USA) was subjected to the 1.0 W plasma jet at 30 °C. Unless otherwise stated, the results presented in this paper are based on the 8.0 W plasma jet apparatus.

The H_2_O_2_ concentration in the solution after CAP irradiation was estimated using a colorimetric staining probe (WAK-H_2_O_2_; Kyoritsu Chemical-Check Laboratory, Yokohama, Japan) in the presence and absence of 100 μg/mL of catalase.

### 3.3. NMR Experiments

NMR spectral measurements were made on a Bruker DMX-500 spectrometer equipped with a cryogenic probe (Bruker, Billerica, MA, USA). ^1^H–^15^N HSQC spectra were measured at 5 °C with 0.1 mM [U-^15^N] Aβ (1–40) solution with 5% (*v*/*v*) ^2^H_2_O with and without pretreatment with CAP or in the presence and absence of varying amounts of H_2_O_2_. The samples were stored at 37 °C during time-course NMR measurements.

### 3.4. HPLC Analyses

The Aβ(1–40) samples with and without plasma irradiation were analyzed by the HPLC system (Shimazu, Kyoto, Japan) using a reverse-phase C18 column (Sunniest C18; ChromaNik Technologies Inc., Osaka, Japan) with a water:acetonitrile-0.1%(*v*/*v*) trifluoroacetic acid gradient at a flow rate of 1.0 mL/min. Each fraction containing Aβ (1–40) was collected and lyophilized for subsequent analyses.

### 3.5. MS Analyses

The determination of the molecular weight of Aβ (1–40) was performed using MALDI-TOF-MS. The samples were mixed with the 2,5-dihydroxy-benzoic acid (DHBA) matrix solution and then detected by a MALDI-TOF high-resolution mass spectrometer (microflex LRF; Bruker Daltonics, Billerica, MA, USA) under negative ion mode.

LC-MS/MS analysis was performed on the lyophilized fractions of [U-^13^C, ^15^N] Aβ (1–40), separated by a reverse-phase C18 column (Sunniest C18; ChromaNik Technologies Inc.). The lyophilized powder was dissolved in 40 mM NH_4_HCO_3_/10% (*v*/*v*) acetonitrile and incubated with trypsin (MS-grade Trypsin Gold; Promega, Madison, WI, USA) at 37 °C for 2 h. MS spectra were acquired using a LC-MS (SCIENTIFIC Orbitrap Elite; Thermo Fisher Scientific, Waltham, MA, USA). The MS/MS data were acquired and processed using the MassLynx software (Waters, Milford, MA, USA), and MASCOT (Matrix Science, London, UK) was used to search the database.

### 3.6. ThT Assay

Aβ (1–40) (0.1 mM) and Aβ (1–42) (0.05 mM) solutions were supplemented with 200 μM ThT from a 2 mM stock solution. Each sample was then pipetted into the multiple wells of a 96-well half-area, low-binding polyethylene glycol coating plate (Corning 3881) with a clear bottom, at 80 μL per well, followed by incubation at 37 °C under quiescent conditions in a plate reader (Infinite 200Pro; TECAN, Zurich, Switzerland). The ThT fluorescence was measured through the bottom of the plate with a 430 nm excitation filter and a 485 nm emission filter. The ThT fluorescence was followed for three repeats of each sample.

### 3.7. TEM

Amyloid fibrils of Aβ (1–40) proteins were subjected to negative stain EM analyses. Briefly, the specimens stained with 2% uranyl acetate on the grid were observed with a 80 kV electron microscope (JEM-1010; JEOL Inc., Tokyo, Japan). The fibril images were collected at a nominal magnification of 100,000×.

## 4. Conclusions

In the present study based on NMR, MS, and kinetics analyses, we clearly demonstrated that the CAP irradiation results in selective oxidation of Met35 of Aβ (1–40), which suppresses amyloid fibril formation. This modification is made by reactive oxygen species generated in the plasma-irradiated buffer solution, rather than by the direct action of the plasma. Indeed, our results are in qualitative agreement with experiments in which H_2_O_2_ was used for oxidation of Aβ, which strongly suggests that H_2_O_2_ is a potent candidate responsible for the Aβ oxidation and the consequent inhibition of its fibril formation. We also suggest that the degradation of Aβ fibrils on CAP irradiation is, at least partially, ascribed to H_2_O_2_-mediated Met35 oxidation. It has been reported that the oligomer formation of Aβ is attenuated by methionine oxidization [29] and the methionine oxidized form of Aβ has low toxicity [30,31]. Therefore, it can be expected that Aβ (1–40) prepared by plasma irradiation is also less toxic. The elucidation of the molecular mechanisms behind the effects of plasma irradiation on amyloid formation provide fundamental insights into plasma biology, giving clues for developing novel applications of CAP in the fields of materials and medical sciences.

## Data Availability

The data presented in this study are available on request from the corresponding author.

## Supplementary Materials

Supplementary materials can be found at https://www.mdpi.com/1422-0067/22/6/3116/s1.
