# Supplementary material for: Cold Atmospheric Plasma Modification of Amyloid β
Source: Int J Mol Sci. 2021 Mar 18;22(6):3116. doi: 10.3390/ijms22063116 (PMC8003251; doi:10.3390/ijms22063116)
Supplement: Supplementary file 1 [file ijms-22-03116-s001.pdf]

Supplementary Material

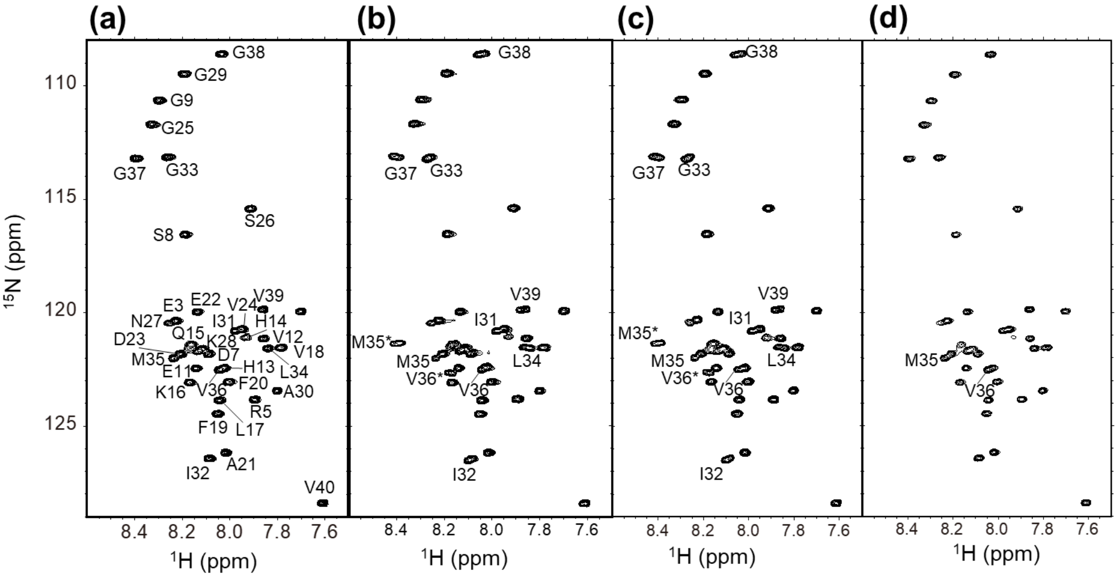

Figure S1. NMR spectral changes of CAP-treated Aβ(1–40). (**a**) ^{1}H-^{15}N HSQC spectrum of Aβ(1–40) without irradiation. ^{1}H-^{15}N HSQC spectra of Aβ(1–40) measured at 48 h (**b**) after a 10-s irradiation of CAP or (**c**) after dissolving into the 10-s-pre-irradiated buffer solution. (**d**) ^{1}H-^{15}N HSQC spectrum of Aβ(1–40) with a 10-s-CAP irradiation in the presence of 1 mM ascorbic acid.

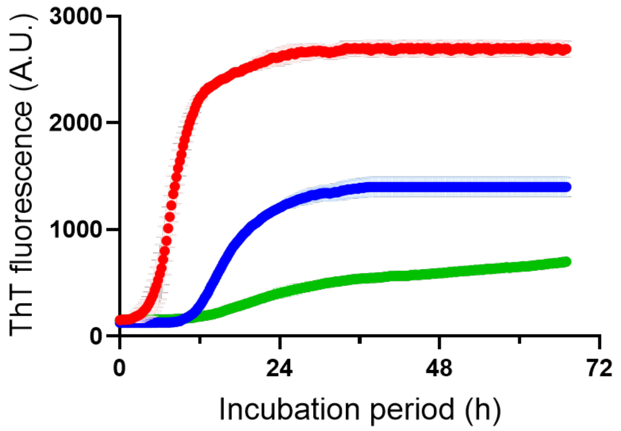

Figure S2. ThT fluorescence intensity profiles of the aggregation of Aβ(1–42) without (red) and with CAP pretreatment for 10 s (blue) or 20 s (green). Each intensity value is the mean ± S.D. of three values.

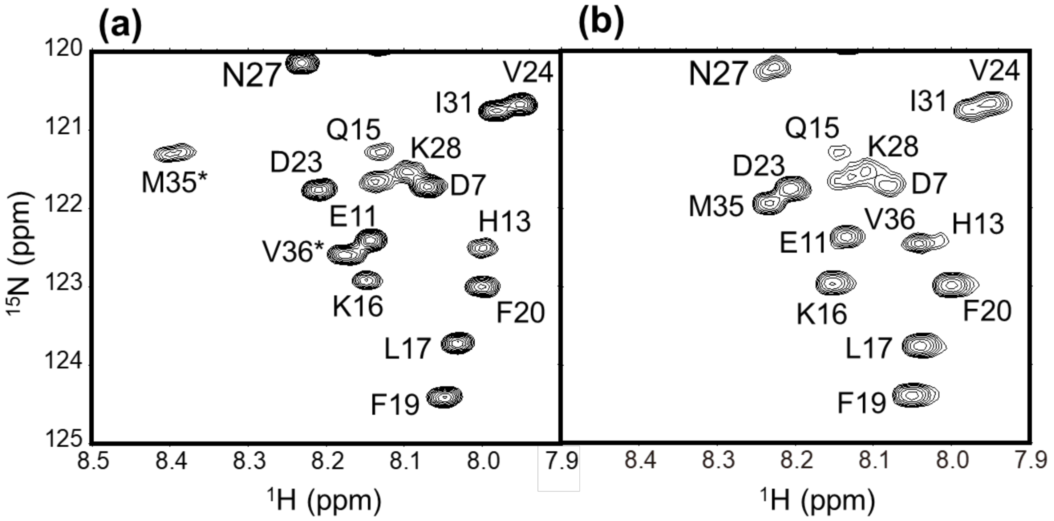

Figure S3. ^{1}H-^{15}N HSQC spectral comparison between Aβ(1–40) species corresponding to (**a**) peak-1 and (**b**) peak-2.

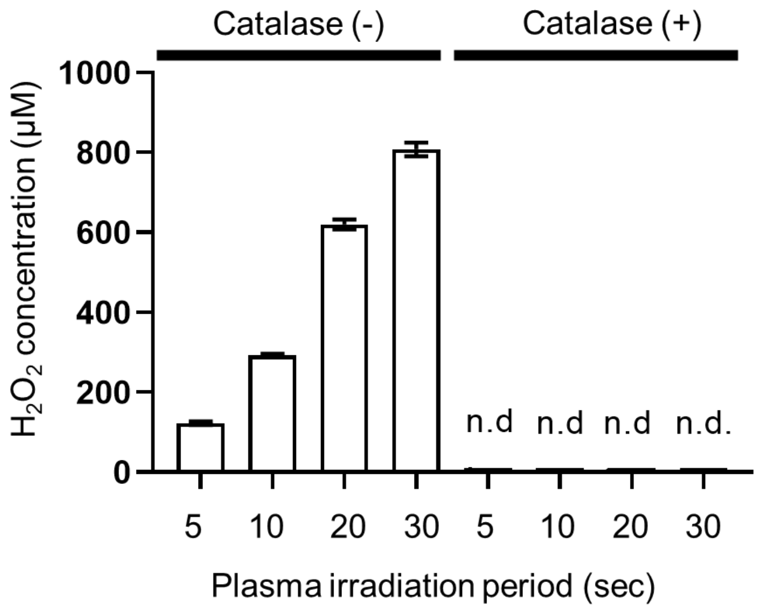

Figure S4. H_2O_2 concentration generated in CAP-irradiated buffer depending on the irradiation period in the absence and presence of 100 μg/mL of catalase using the 8.0 W-plasma jet. Error bars represent the standard error of the mean ($n = 3$ independent CAP-irradiation).

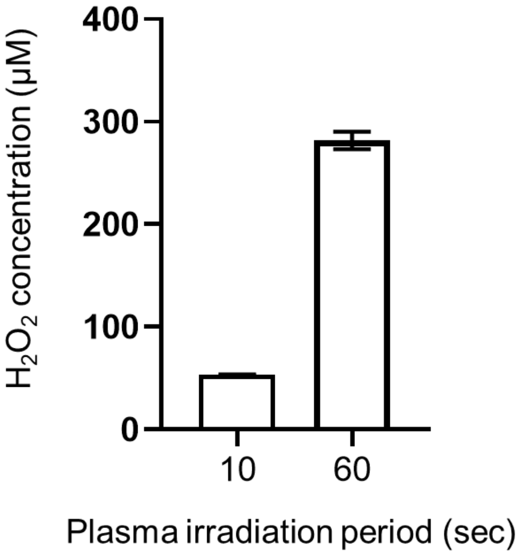

Figure S5. H_2O_2 concentration generated in CAP-irradiated buffer depending on the irradiation period using the 1.0 W-plasma jet. Error bars represent the standard error of the mean ($n = 3$ independent CAP-irradiation).

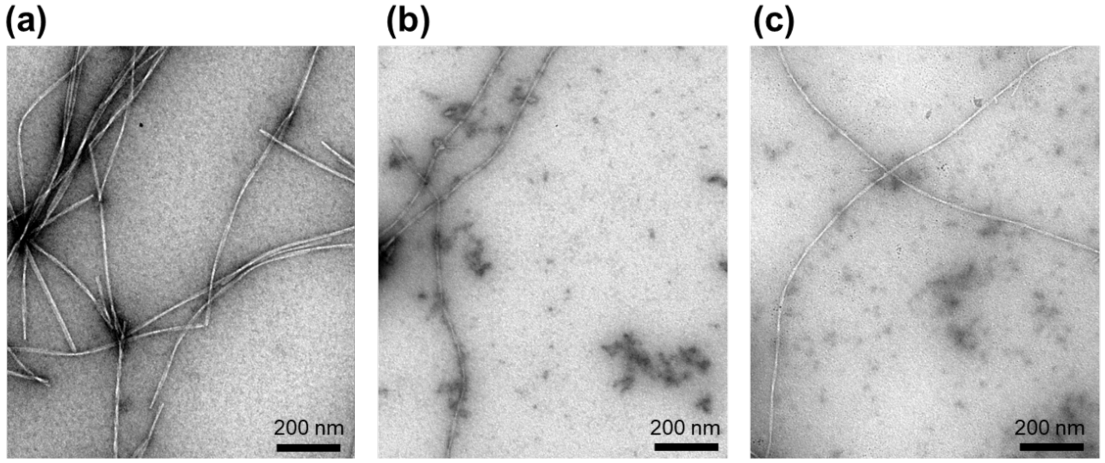

Figure S6. TEM image of Aβ(1–40) fibrils. (**a**) Fibrils without CAP irradiation, (**b**) fibrils incubated for 24 h at 37°C in the 30-s-CAP-pre-irradiated buffer, and (**c**) fibrils treated with 800 μM of H_2O_2 for 24 h at 37°C.

Table S1. LC-MS/MS MASCOT results of tryptic digestions of the CAP-irradiated Aβ(1–40) fraction.

m/z	*z*	Observed mass	Calculated mass	Ion score	Peptide sequence[a]
581.8848	2	1161.7551	1161.7552	60.20	GAIIGLM*VGGVV
581.8850	2	1161.7555	1161.7552	53.30	GAIIGLM*VGGVV
581.8851	2	1161.7556	1161.7552	60.57	GAIIGLM*VGGVV
581.8851	2	1161.7556	1161.7552	55.85	GAIIGLM*VGGVV
581.8851	2	1161.7557	1161.7552	44.17	GAIIGLM*VGGVV
581.8853	2	1161.7559	1161.7552	57.67	GAIIGLM*VGGVV
581.8854	2	1161.7562	1161.7552	58.48	GAIIGLM*VGGVV
446.2064	3	1335.5973	1335.5956	28.32	HDSGYEVHHQK
706.8718	2	1411.7290	1411.7305	50.09	HDSGYEVHHQK
353.9397	4	1411.7298	1411.7305	39.73	HDSGYEVHHQK
706.8725	2	1411.7304	1411.7305	39.08	HDSGYEVHHQK
706.8726	2	1411.7307	1411.7305	40.19	HDSGYEVHHQK
471.5842	3	1411.7308	1411.7305	24.75	HDSGYEVHHQK
706.8729	2	1411.7312	1411.7305	44.67	HDSGYEVHHQK
706.8729	2	1411.7312	1411.7305	41.03	HDSGYEVHHQK
706.8729	2	1411.7313	1411.7305	42.91	HDSGYEVHHQK
471.5844	3	1411.7315	1411.7305	39.49	HDSGYEVHHQK
706.8731	2	1411.7315	1411.7305	57.54	HDSGYEVHHQK
706.8732	2	1411.7318	1411.7305	39.34	HDSGYEVHHQK
706.8733	2	1411.7321	1411.7305	43.95	HDSGYEVHHQK
706.8735	2	1411.7325	1411.7305	31.99	HDSGYEVHHQK
706.8735	2	1411.7325	1411.7305	43.14	HDSGYEVHHQK
706.8737	2	1411.7329	1411.7305	25.30	HDSGYEVHHQK
706.8737	2	1411.7329	1411.7305	37.91	HDSGYEVHHQK
706.8741	2	1411.7337	1411.7305	22.34	HDSGYEVHHQK
689.3646	3	2065.0719	2065.0735	24.08	DAEFRHDSGYEVHHQK
689.3646	3	2065.0720	2065.0735	22.07	DAEFRHDSGYEVHHQK
689.3647	3	2065.0724	2065.0735	21.80	DAEFRHDSGYEVHHQK
689.3649	3	2065.0728	2065.0735	25.51	DAEFRHDSGYEVHHQK
689.3649	3	2065.0728	2065.0735	41.72	DAEFRHDSGYEVHHQK
689.3652	3	2065.0739	2065.0735	46.22	DAEFRHDSGYEVHHQK
689.3655	3	2065.0748	2065.0735	33.33	DAEFRHDSGYEVHHQK
689.3657	3	2065.0753	2065.0735	23.55	DAEFRHDSGYEVHHQK
689.3658	3	2065.0755	2065.0735	36.49	DAEFRHDSGYEVHHQK
689.3659	3	2065.0759	2065.0735	18.89	DAEFRHDSGYEVHHQK
689.3660	3	2065.0761	2065.0735	35.24	DAEFRHDSGYEVHHQK
689.3661	3	2065.0764	2065.0735	24.64	DAEFRHDSGYEVHHQK
559.9392	3	1676.7956	1676.7947	28.44	FNWYVDGVEVHNAK

[a] M* denotes oxidized Met.
